# Effects of Carbon Black and the Presence of Static Mechanical Strain on the Swelling of Elastomers in Solvent

**DOI:** 10.3390/ma8030884

**Published:** 2015-03-02

**Authors:** Shiau Ying Ch’ng, Andri Andriyana, Yun Lu Tee, Erwan Verron

**Affiliations:** 1 Department of Mechanical Engineering, Faculty of Engineering, University of Malaya, Kuala Lumpur 50603, Malaysia; E-Mail: yunlu90@siswa.um.edu.my; 2 Taylor’s University, Taylor’s Lakeside Campus, No.1, Jalan Taylor’s, Subang Jaya 47500, Selangor Darul Ehsan, Malaysia; E-Mail: shiauying.chng@taylors.edu.my; 3 LUNAM Université, École Centrale de Nantes, GeM, UMR CNRS 6183, BP 92101, Nantes 44321, France; E-Mail: erwan.verron@ec-nantes.fr

**Keywords:** elastomers, swelling, carbon black, mechanical loading, large deformation

## Abstract

The effect of carbon black on the mechanical properties of elastomers is of great interest, because the filler is one of principal ingredients for the manufacturing of rubber products. While fillers can be used to enhance the properties of elastomers, including stress-free swelling resistance in solvent, it is widely known that the introduction of fillers yields significant inelastic responses of elastomers under cyclic mechanical loading, such as stress-softening, hysteresis and permanent set. When a filled elastomer is under mechanical deformation, the filler acts as a strain amplifier in the rubber matrix. Since the matrix local strain has a profound effect on the material’s ability to absorb solvent, the study of the effect of carbon black content on the swelling characteristics of elastomeric components exposed to solvent in the presence of mechanical deformation is a prerequisite for durability analysis. The aim of this study is to investigate the effect of carbon black content on the swelling of elastomers in solvent in the presence of static mechanical strains: simple extension and simple torsion. Three different types of elastomers are considered: unfilled, filled with 33 phr (parts per hundred) and 66 phr of carbon black. The peculiar role of carbon black on the swelling characteristics of elastomers in solvent in the presence of mechanical strain is explored.

## 1. Introduction

Rubber products are common in our daily life and, in fact, are one of the essential materials used in many industrial applications. However, the original rubber in latex form is not practical unless it is processed with vulcanization and compounded with additives [1]. The major additives used in rubber compounds include fillers, antioxidants, antiozonants, curing agents and processing aids. It is well known that the cost of rubber products can be lowered and the mechanical properties, such as stiffness, modulus, tear resistance, tensile strength and fatigue resistance, can be significantly improved by adding filler [2,3]. While unfilled rubbers exhibit nearly ideal nonlinear elastic behavior, the incorporation of filler alters the stress-softening behavior, known as the Mullins effect, and the time-dependent behavior characterized by hysteresis and stress relaxation [4].

During their applications, rubber products are frequently subjected to various hostile environments, e.g., aggressive solvents, in addition to fluctuating mechanical loading. Such conditions may affect the service life of elastomers by two major factors: by fatigue due to fluctuating mechanical loading and by swelling due to the diffusion of solvents [5]. While numerous results corresponding to free swelling are available in the literature, the extension to the case where elastomers are simultaneously subjected to mechanical loading is less common [6], not to mention the case involving different amounts of carbon black content.

When elastomers are exposed to aggressive liquids, material degradation in the form of swelling occurs [7,8]. With swelling, the liquids occupy positions among the polymer molecules resulting to the increase in chain separation and, thus, decreasing the intermolecular forces. Consequently, the material becomes softer. While the precise effect of the presence of mechanical loading is unknown, it appears that mechanical loading significantly influences the swelling characteristics of elastomers in solvent [6,9,10].

The main interest of this work is to investigate the effect of carbon black content and mechanical loading on the swelling characteristics of elastomers in solvent. For this purpose, special devices are developed, such that the immersion tests can be conducted on rubber specimens simultaneously undergoing static uniaxial or multiaxial mechanical loadings. The information of the materials and the description of the experimental procedures are provided in Section 2. The results are presented and discussed in Section 3, while concluding remarks are given in Section 4.

## 2. Experimental Section

### 2.1. Materials and Specimens

Two different specimen shapes are used for investigating the effect of carbon black content and mechanical loading on the swelling of elastomers. The as-received elastomeric specimens were provided by Malaysia Rubber Board. The specimens are vulcanized by performing compression molding at 165 °C for 5 min under a pressure of approximately 6.89 MPa in an electrically-heated press. Due to confidentiality issues, the detailed compositions are not provided in this paper. Instead, the elastomeric specimens are classified by the amount of carbon black in the compositions.

**Figure 1.**
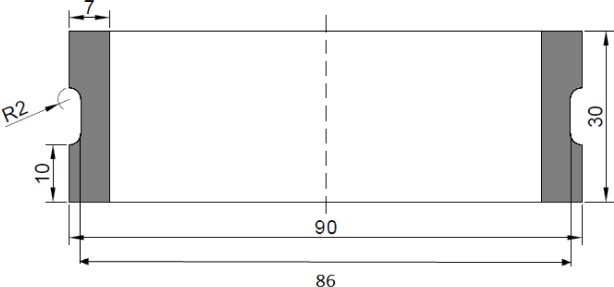
Detailed dimensions of the cylindrical hollow specimen for multiaxial mechanical loading.

To probe the effect of carbon black content, dumbbell elastomeric specimens following ASTM standard D412-C are used. The materials include unfilled nitrile butadiene rubber (NBR) and filled NBR with 33 phr and 66 phr of carbon black, respectively. In order to investigate the effect of the presence of static mechanical deformation, specially designed cylindrical hollow elastomeric specimens with 33 phr of carbon black are used. The detailed dimensions of the cylindrical hollow elastomeric specimen are given in Figure 1. Note that the latter is an improved version of the one proposed in our previous work [5]. The solvent used for immersion tests is 100% palm biodiesel and is referred to as B100 in the following. The tests are conducted under room temperature for different immersion durations. The detailed chemical composition of the B100 used in the present study is provided in our previous work [6] and will not be recalled here.

### 2.2. Experimental Devices and Procedures

**Figure 2.**
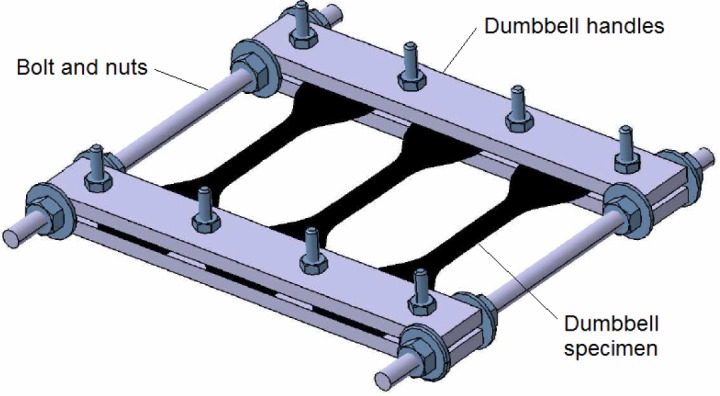
Specially-designed device for the immersion test of rubber specimens in the presence of uniaxial mechanical loading.

**Figure 3.**
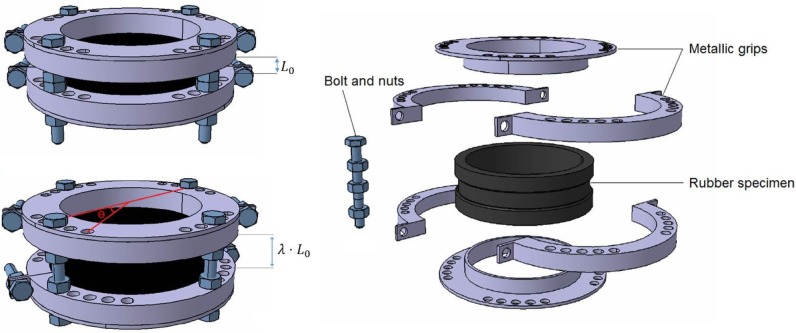
Specially-designed device for the immersion test of rubber specimens in the presence of multiaxial mechanical loading.

Two different devices are developed to enable the application of static uniaxial and multiaxial strains on the specimens during the immersion tests. They are illustrated in Figures 2 and 3, where dumbbell and cylindrical hollow elastomeric specimens are used, respectively. The description of the device and experimental procedure for the uniaxial device can be summarized as follows:

1. The device consists of four identical metallic plates (handles) and two long bolts. Each plate has four holes. The device can accommodate three dumbbell specimens.
2. Two plates are attached to the upper and to the lower parts of the specimens. Bolts are inserted into the three holes located on the plates. The plates are then tightened using nuts. While doing this procedure, the long bolts are fit into each side of the plates.
3. Tensile strain can be applied by adjusting the nuts located at the long bolts between metallic plates until the desired strain level is achieved.
4. The whole device containing stretched specimens is left untouched under room temperature for 24 h prior to the immersion in B100 to allow for stress relaxation in the elastomeric specimen in order to eliminate the viscoelastic behavior.

In the case of multiaxial loading as depicted in Figure 3, the description of the device and experimental procedure can be summarized as follows:

1. The device consists of two identical circular metallic plates and four identical semi-circular metallic grips. Each plate and grip has sixteen and eight holes, respectively, located at an angle of 10° apart from each other. Moreover, each side of the grip contains one additional hole. The device can accommodate one cylindrical hollow specimen.
2. The plates and grips are attached to the specimen. Bolts and nuts are used on each side of the grips in order to tighten the device.
3. The specimen in the device is twisted. The twist angle is held constant by inserting bolts into holes located on the grips and plates. Tensile strain can be applied by adjusting the nuts. Thus, the specimen is subjected to simultaneous tensile and torsion loadings.
4. The whole device containing the twisted/stretched specimen is left untouched under room temperature for 24 h prior to the immersion in B100 to allow for stress relaxation in the elastomeric specimen in order to eliminate the viscoelastic behavior.

After a certain immersion duration, the elastomeric specimens are dismantled from the device, and their volume is measured in order to determine the degree of swelling undergone by the materials. It is noted that different sets of devices/specimens are used for different immersion durations. For each immersion duration and each level of imposed strain, at least three specimens are used, and the presented swelling data correspond to the average value. The percentage of volume change is calculated using the following simple relation [11]:

(1) $$\%\;Volume\;Change=\frac{\left(M_{2}-M_{4})-(M_{1}-M_{3}\right)}{\left(M_{1}-M_{3}\right)}\times 100$$

where M_1_ and M_2_ are the masses in air (grams) before and after immersion, while M_3_ and M_4_ are the masses in water (grams) before and after immersion.

**Remark 1.** *It is noted that the calculation of the volume change based on Equation (1) is taken from the whole specimen, including the clamping area. The calculation is corrected based on the exposed volume by assuming that there is no swelling occurring in the clamping area during the immersion and after the removal from the experimental device.*

## 3. Results and Discussion

### 3.1. Stress-Free Swelling

**Figure 4.**
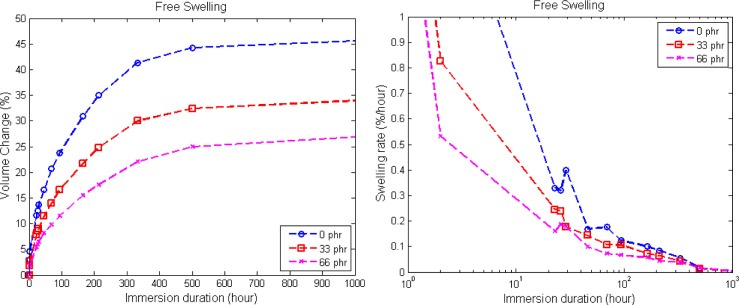
Volume change undergone by the specimens with different carbon black contents **(a)** and the corresponding rates of swelling **(b)**.

In order to ensure a stress-free state in the dumbbell rubber specimens during immersion testing, the distance between the metallic handles is adjusted from time to time, so that the specimens are free to swell without buckling. Figure 4 shows the resulting volume change from dry until equilibrium swelling and the resulting rates of swelling. From the figure, it is observed that the unfilled elastomer has the highest volume change percentage, followed by 33 phr and 66 phr of carbon black content, respectively. The presence of carbon black appears to restrict the diffusion of solvent into the elastomers, since the elastomeric network becomes stiffer with the addition of filler [3]. Thus, it provides more resistance and a barrier to solvent penetration [12]. Physically, the filled elastomer with 66 phr of carbon black is the stiffest. For the unfilled specimen, the diffusion of solvent molecules into the elastomeric matrix is favorable, as there is no filler preventing the diffusion of solvent into the free volume between the molecules [13]. Regarding the rate of swelling, it is relatively high in the beginning of the immersion, as there is a high concentration gradient between the dry elastomer and biodiesel. Subsequently, the rate of swelling decreases with the increase in immersion duration and approaches zero as the material reaches equilibrium swelling. As indicated in Figure 4, the introduction of filler appears to restrict the rate of swelling [14].

**Figure 5.**
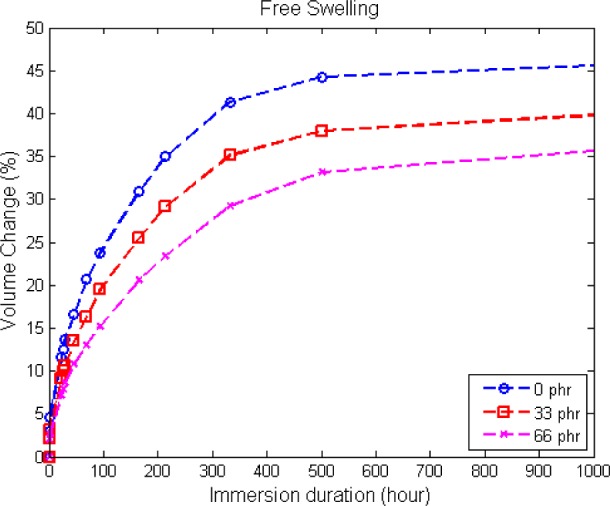
Corrected volume change undergone by the specimens with different carbon black contents.

In the case where the filler is considered as rigid particle and does not participate in the swelling process, swelling only occurs in the rubber matrix, and the measured volume changes need to be corrected based on the matrix:filler ratio. The result is presented in Figure 5. The result is unaffected for unfilled rubber, since the specimen is composed of 100% rubber matrix, but higher swelling is obtained for filled rubber. Overall, a similar trend is observed where a higher amount of carbon black leads to lower swelling. However, note that the difference in volume changes between each carbon black content is significantly smaller for the corrected results.

## 3.2. Constrained Swelling 1: Swelling in the Presence of Static Uniaxial Strain

**Figure 6.**
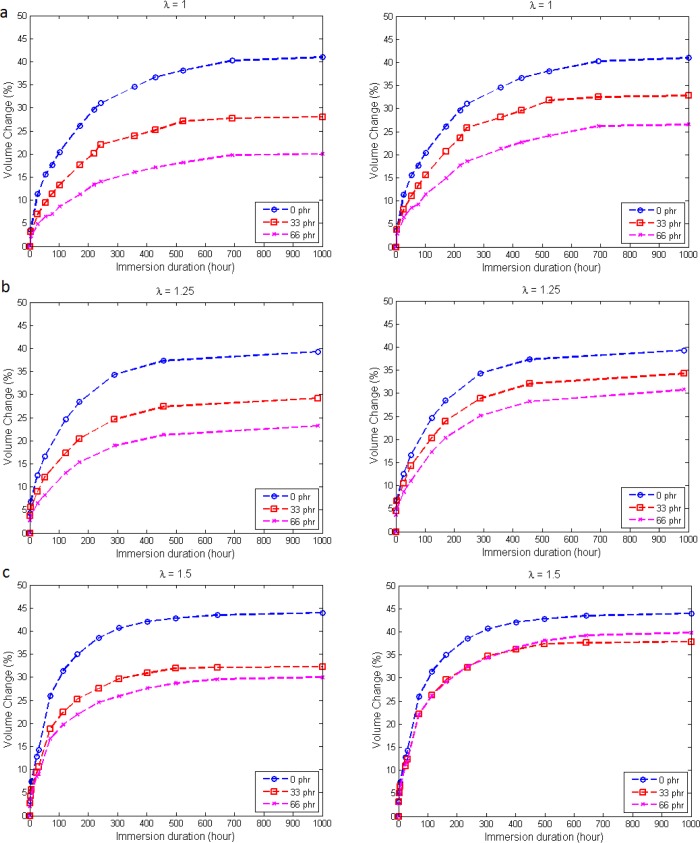
Measured volume change (left) and corrected volume change (right) of specimens for different carbon black contents at **(a)** λ = 1; **(b)** λ = 1.25 and **(c)** λ = 1.5.

In this section, the effect of the presence of static uniaxial mechanical strain on swelling is addressed. For this purpose, the distance between the metallic handles are fixed at three different constant stretches: λ = 1.00, 1.25 and 1.50, respectively. The stretch is defined as the ratio between the current length to the original length of the specimen between the two metallic handles. Note that the corresponding engineering strain is simply given by *ε* = λ − 1. The resulting measured volume change percentage and corrected volume change percentage based on the matrix:filler ratio are given in Figure 6.

Generally, for all imposed strain levels, the swelling trend is similar to the one for free swelling, where the unfilled rubber specimens exhibit the highest swelling level, followed by rubber specimens with 33 phr and with 66 phr of carbon black. Moreover, the introduction of static uniaxial strain appears to alter the swelling characteristics of elastomers [15]. For a given immersion duration, uniaxial tensile strain acts as the accelerator for the penetration of solvent molecules, *i.e.*, increasing the rate of swelling and, thus, increasing swelling.

The increasing swelling level with strain can be explained by the hydrostatic part of Cauchy stress in the elastomer. According to [8], negative hydrostatic stress facilitates swelling, and the application of tensile strain generates more negative hydrostatic stress. Thus, the resulting swelling increases as more tensile strain is applied to the specimen.

For filled elastomers, closer investigation in Figure 6 reveals that the swelling curve of 33 phr carbon black and the one of 66 phr carbon black become closer as a higher strain is applied. This observation could be related to two opposite roles of the filler on the ability of elastomers to absorb solvent. On the one hand, as discussed in the previous section, increasing carbon black content yields a stiffer elastomeric network, which acts as a barrier to solvent penetration [16]. On the other hand, carbon black acts as a strain amplifier when a macroscopic strain is applied to the elastomers [17]: the higher the carbon black content, the higher the strain amplification. The strain amplifying concept considers carbon black as a rigid particle that does not participate in the deformation when a strain is applied to the elastomer. The soft rubbery matrix is assumed to behave exactly the same as the pure vulcanizate without the carbon black [18]. As a result, the local strain in the elastomer is greater than the macroscopic applied strain due to the amplified strain effect. This, in turn, will generate more negative hydrostatic stress, which facilitates solvent penetration.

As for the corrected volume changes presented on the right-hand side of Figure 6, a similar trend as stress-free immersion is observed here, where the difference between each carbon black content reduces, and this phenomenon is more obvious at higher strain levels. In fact, a special case is observed at λ = 1.5, where the volume change for rubber specimen with 66 phr of carbon black overtakes the one with 33 phr of carbon black. This observation, which is caused by the strain amplification effect as explained earlier, serves as an important factor in formulating the rubber compounds. While filler is known to restrict swelling, the combination of filler amount and tensile strain needs to be taken into consideration in order to obtain a rubber component that is susceptible to solvent and simultaneously subjected to tensile strain.

### 3.3. Constrained Swelling 2: Swelling in the Presence of Static Multiaxial Strain

**Figure 7.**
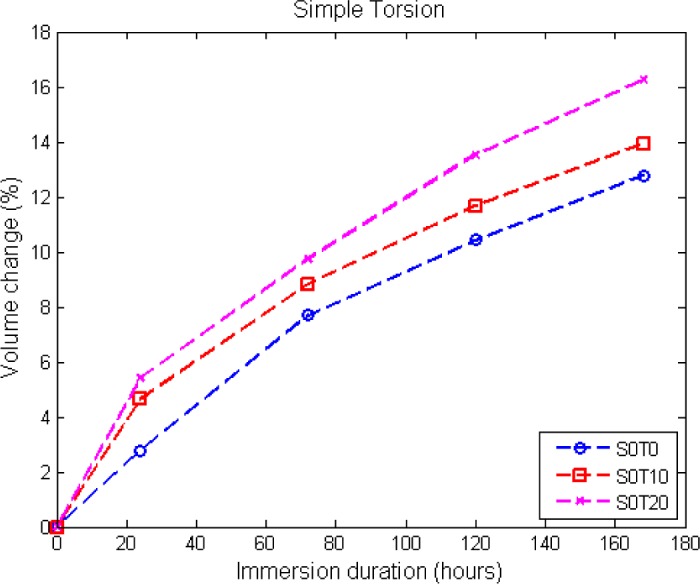
Volume change for different twisting angles.

In this section, the effect of static multiaxial strain on the swelling characteristics of elastomers is explored. For this purpose, only elastomers with 33 phr of carbon black are considered. Three different twist angles are imposed: 0°, 10° and 20°. The details of the applied mechanical deformations are summarized in Table 1, and the resulting volume change is shown in Figure 7.

As shown in Figure 7, the introduction of torsional strain yields a higher volume change compared to the initially stress-free specimen. The volume change also increases with the increasing in immersion duration. At the end of immersion (170 h), the percentages of volume change are approximately 13%, 14% and 16.3% for twist angles of 0° (initially stress-free), 10° and 20°, respectively. However, much to our disappointment, the percentage of volume change at equilibrium swelling could not be obtained with this design, since the specimen breaks after 170 h of immersion.

### 3.4. Hydrostatic Part of Cauchy Stress as a Predictor

According to [8], the amount of swelling in a material is governed by the hydrostatic part of Cauchy stress generated as a result of the applied mechanical loading. By utilizing this theory, the hydrostatic part of Cauchy stress for different mechanical loadings is calculated and used as a predictor for swelling.

**Figure 8.**
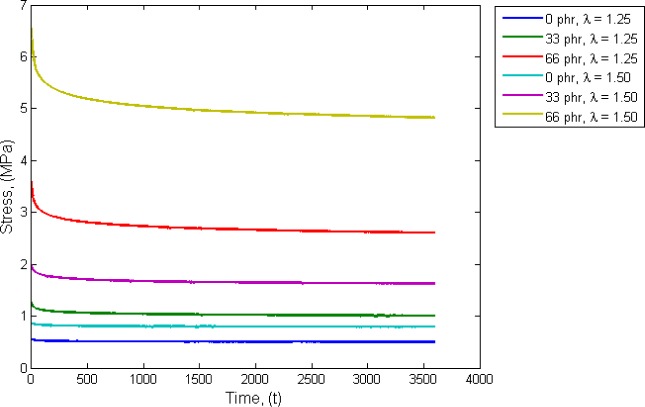
Stress relaxation for unfilled and filled dumbbells at different strain levels.

First, consider the simple case of uniaxial load. In order to probe for the viscoelastic response of the materials, a set of relaxation tests is conducted on dumbbell specimens at two different stretches. The results are presented in Figure 8. In this figure, it is clearly shown that the stress relaxation (viscoelasticity) becomes significant as the filler content increases. In this case, the calculation of the hydrostatic stress will be based on the relaxed state of stress.

**Figure 9.**
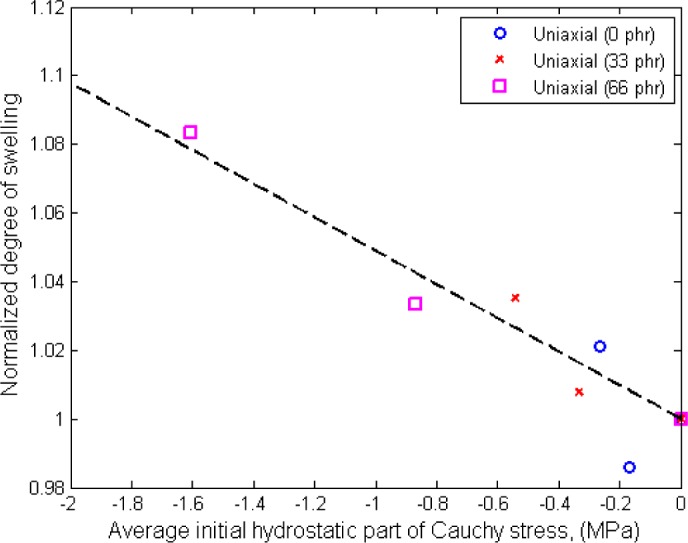
Normalized degree of swelling as a function of hydrostatic stress.

The hydrostatic stress is obtained by taking the trace of the Cauchy stress tensor [19]:

(2) $$p=-\frac{1}{3}(\mathrm{tr\sigma})$$

which simplifies to:

(3) $$p=-\frac{1}{3}(\sigma_{11})$$

for the case of uniaxial loading. Using the result obtained from relaxation tests, the hydrostatic stresses calculated from Equation (3) are plotted against their corresponding normalized degree of swelling in Figure 9. For each carbon content, the normalized degree of swelling is defined by the ratio between the swelling of strained specimen over the one of stress-free specimen (free swelling).

From Figure 9, the efficiency of hydrostatic stress as a predictor for swelling is clearly illustrated. The normalized degree of swelling increases as the value of hydrostatic stress becomes more negative. In other words, negative hydrostatic stress generates a tensile state in the specimen that provokes the diffusion of solvent into the specimen. Thus, the resulting degree of swelling at equilibrium is higher. As seen in Figure 9, regardless of the amount of applied tensile strain and the filler content in the specimen, the normalized degree of swelling appears to be a linear function with the resulting hydrostatic stress in the specimen.

**Remark 2.** *The filled elastomers used in the present study show a strong viscoelastic response. Thus, under static torsion, the resulting hydrostatic stress evolves with time. Since the precise kinetics of stress relaxation in the material under torsion during immersion testing is not known, the hydrostatic stress used here is estimated by calculating the initial hydrostatic stress in the dry specimen before the immersion test.*

**Figure 10.**
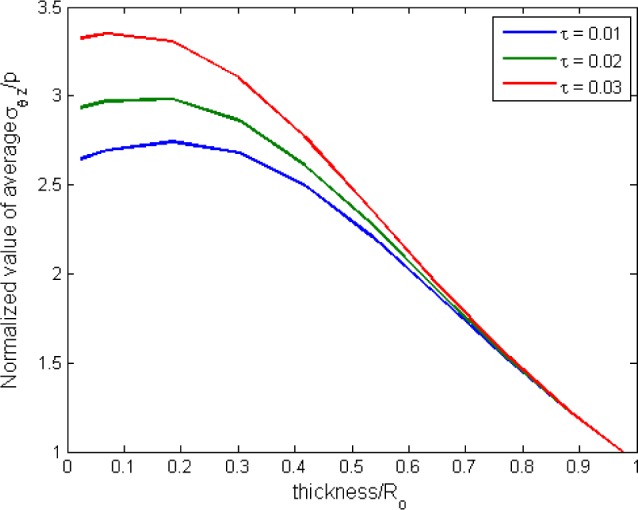
Normalized ratio of ${\bar{\sigma }}_{\theta z}/\bar{p}$ as a function of thickness/*R_o_*.

Next, for the case of hollow cylinder with inner radius *R_i_* and outer radius *R_o_* under torsion, the deformation is more complex, and it can be described using a cylindrical coordinate system. The detailed derivation of the hydrostatic stress in the hyperelastic tube under torsion is given in Appendix A. Since torsional loading generates shear stress in the material, the normalized values of the ratio between the average shear stress and the average hydrostatic stress are plotted against the ratio of cylinder thickness to *R_o_* in Figure 10 for three different values of twist. For each twist, the ${\bar{\sigma }}_{\theta z}/\bar{p}$ is normalized by its value for a solid cylinder. Moreover, the average value is calculated using the following simple equation:

(4) $$\begin{array}{l} \bar{p}=\frac{1}{R_{o}-R_{i}}\int_{R_{i}}^{R_{o}}p(R)\cdot dR\\ {\bar{\sigma }}_{\theta z}=\frac{1}{R_{o}-R_{i}}\int_{R_{i}}^{R_{o}}\sigma_{\theta z}(R)\cdot dR \end{array}$$

From Figure 10, it is clearly illustrated that for higher values of thickness/*R_o_* (*i.e.*, thick wall cylinder or solid cylinder for the case of thickness/*R_o_* = 1), the normalized ratio of shear stress to the hydrostatic stress is close to unity and does not show significant dependence on the amount of twist angle. However, as the value of thickness/*R_o_* decreases (*i.e.*, approaching a thin wall cylinder), the normalized value becomes highly dependent on the amount of the twist angle. Moreover, the contribution of shear stress becomes predominant as compared to hydrostatic stress. Based on this observation, it is shown that for a thin wall cylinder under torsional loading, the amount of shear stress generated contributes a significant part to the stress tensor.

Using the results obtained in Figure 10, a discussion based on the specimen used in this research is highlighted here. The specimen used in this research has dimensions as shown in Figure 1, which corresponded to a thin wall cylinder with thickness/*R_o_* = 0.12. From Figure 10, the normalized value of the shear stress to the hydrostatic stress increases as the amount of the twist angle increases for thickness/*R_o_* = 0.12. As for the experimental observation, it is found that the degree of swelling increases with torsional loading. Thus, our observation suggests that the degree of swelling for a thin wall cylinder increases with the normalized value of the shear stress to the hydrostatic stress. However, this cannot be fully validated, since the specimens underwent rupture before any equilibrium swelling could be achieved. Thus, further specimen improvements are needed in order to overcome this limitation. Moreover, considering the significant contribution of shear stress in the thin-walled cylinder under torsion, pure consideration of hydrostatic stress in the prediction of equilibrium swelling may not be sufficient.

## 4. Conclusions

In this study, two important factors that affect the swelling characteristics of elastomers in solvent were addressed: carbon black filler and the presence of static mechanical loading. For this purpose, original devices and specimens were developed so that the swelling test could be conducted on the specimens while they were simultaneously subjected to static mechanical loading: uniaxial extension and simple torsion. It was found that the introduction of carbon black filler reduces the swelling of elastomers. The carbon black acts as a barrier, which prevents the diffusion of solvent into the elastomeric matrix. However, when a static tensile strain is present, the carbon black acts also as a strain amplifier, which facilitates liquid uptake.

The effect of static uniaxial mechanical loading on swelling can be probed by determining the resulting hydrostatic part of the Cauchy stress. As suggested by [8], negative hydrostatic stress facilitates swelling, while the positive one restricts swelling. The effect of static simple torsion on the swelling characteristics was somewhat more complex, since a significant amount of shear stress is generated. It is suggested that the consideration from purely hydrostatic stress may not be sufficient, especially for the case of a thin wall cylinder. Further improvements on the specimen design are needed to probe a clear conclusion, and works are currently being carried out by the authors.

To close this paper, it is noted that in view of the durability analysis, our findings clearly show the need to develop a mathematical model describing the coupling effect between the diffusion of solvent and mechanical deformation. Such a model is currently being developed by the authors.

## Figures and Tables

**Table 1.** Mechanical loading conditions.

Specimen	Twist Angle (°)	Resulting Twist per Unit Length, *τ* (rad/mm)	Shear Strain	
			Inner Radius	Outer Radius
S0T0	0	0	0	0
S0T10	10	0.01746	0.66348	0.75078
S0T20	20	0.03491	1.32658	1.50113

## Appendix

### Combined Tensile/Torsion of a Hyperelastic Tube

This Appendix summarizes the derivation of the governing equations for the combined tensile/torsion of a cylindrical hyperelastic tube. Note that the readers can refer to the example from [20] or [21] for a detailed derivation. Let (*R*, Θ, *Z*) and (e*_R_*, e_Θ_, e*_Z_*) be the coordinates and the cylindrical unit vectors in the undeformed configuration. Let (*r*, θ, *z*) and (e*_r_*, e_θ_, e*_z_*) denote their counterparts in the deformed configuration. The deformation of a simultaneous tensile/torsion of a circular tube is given by [22]:

(A1) r=r(R)θ=Θ+λτZz=λZ

λ is the stretch ratio and *τ* is the angle of torsion (in rad) per unit undeformed length. The corresponding deformation gradient for the deformation is:

(A2) $$F=\frac{dr}{dR}e_{r}⊗e_{R}+\frac{r}{R}e_{\theta }⊗e_{\Theta }+\lambda \tau re_{\theta }⊗e_{Z}+\lambda e_{z}⊗e_{Z}$$

Let *r_i_* := *r*(*R_i_*) and *r_o_* := *r*(*R_o_*); we have [19]:

(A3) $$r^{2}=r_{i}^{2}+\frac{1}{\lambda }(R^{2}-R_{i}^{2})$$

The left Cauchy–Green strain tensor can be expressed as:

(A4) $$B=\frac{R^{2}}{r^{2}\lambda^{2}}e_{r}⊗e_{r}+(\frac{r^{2}}{R^{2}}+\lambda^{2}\tau^{2}r^{2})e_{\theta }⊗e_{\theta }+\lambda^{2}\tau r(e_{\theta }⊗e_{z}+e_{z}⊗e_{\theta })+\lambda^{2}e_{z}⊗e_{z}$$

and:

(A5) $$I_{1}=\mathrm{trB}=\frac{R^{2}}{r^{2}\lambda^{2}}+\lambda^{2}\tau^{2}r^{2}+\frac{r^{2}}{R^{2}}+\lambda^{2}$$

Assuming the existence of a hyperelastic strain energy density *W*, which depends solely on *I*_1_, the Cauchy stress tensor reduces to:

(A6) $$\sigma =-qI+\frac{\partial W}{\partial I_{1}}B$$

where *q* is a Lagrange multiplier to be determined from equilibrium equations and appropriate boundary conditions. The normal components of Cauchy stress are:

(A7) $$\begin{array}{l} \sigma_{rr}=-q+\frac{\partial W}{\partial I_{1}}{\left(\frac{R}{r\lambda }\right)}^{2}\\ \sigma_{\theta \theta }=-q+\frac{\partial W}{\partial I_{1}}\left(\frac{r^{2}}{R^{2}}+\lambda^{2}\tau^{2}r^{2}\right)\\ \sigma_{zz}=-q+\frac{\partial W}{\partial I_{1}}\lambda^{2} \end{array}$$

and the shear component is:

(A8) $$\sigma_{\theta z}=\frac{\partial W}{\partial I_{1}}\lambda^{2}\tau r$$

The equilibrium equation div **σ** = **0** (in the absence of body forces) leads to:

(A9) $$\frac{d\sigma_{rr}}{dr}+\frac{\left(\sigma_{rr}-\sigma_{\theta \theta }\right)}{r}=0$$

On the inner and outer surface of the cylinder, there is no lateral load. Under such a restriction, the form of the boundary conditions is given as [23],

(A10) σrr|R=Ro=0σrr|R=Ri=0

By applying the boundary condition $\sigma_{rr}{|}_{R=R_{o}}=0$ to Equation (A9), the Lagrange multiplier can be written as a function of *R*:

(A11) $$q(R)=\frac{\partial W}{\partial I_{1}}\frac{R_{2}}{r^{2}\lambda^{2}}-\frac{\partial W}{\partial I_{1}}\int_{R}^{R_{o}}\left(\frac{R^{3}}{r^{4}\lambda^{3}}-\frac{1}{R\lambda }-\lambda \tau^{2}R\right)dR$$

The relation of *r* in Equation (A3) can be solved using the remaining boundary condition $\sigma_{rr}{|}_{R=R_{i}}=0$, and thus, the expression for the associated stresses can be obtained. Once the associated stresses are obtained, the resulting hydrostatic stress is calculated using Equation (2).
